# Role of facial familiarity and emotional expression intensity in ensemble emotion perception

**DOI:** 10.3758/s13414-023-02720-6

**Published:** 2023-05-22

**Authors:** Deema Awad, Nathan J. Emery, Isabelle Mareschal

**Affiliations:** 1 Department of Psychological and Behavioural Science, London School of Economics, Houghton St, London, London, WC2A 2AE UK; 2 Department of Biological & Experimental Psychology, School of Biological & Chemical Sciences, Queen Mary University of London, London, E1 4NS UK

**Keywords:** Face perception, Familiarity, Ensemble perception, Emotions

## Abstract

When looking at groups of people, we can extract information from the different faces to derive properties of the group, such as its average facial emotion, although how this average is computed remains a matter of debate. Here, we examined whether our participants’ personal familiarity with the faces in the group, as well as the intensity of the facial expressions, biased ensemble perception. Participants judged the average emotional expression of ensembles of four different identities whose expressions depicted either neutral, angry, or happy emotions. For the angry and happy expressions, the intensity of the emotion could be either low (e.g., slightly happy) or high (very happy). When all the identities in the ensemble were unfamiliar, the presence of any high intensity emotional face biased ensemble perception towards its emotion. However, when a familiar face was present in the ensemble, perception was biased towards the familiar face’s emotion regardless of its intensity. These findings reveal that how we perceive the average emotion of a group is influenced by both the emotional intensity and familiarity of the faces comprising the group, supporting the idea that different faces may be weighted differently in ensemble perception. These findings have important implications for the judgements we make about a group’s overall emotional state may be biased by individuals within the group.

## Public significance statement

When looking at a group of people, we can rapidly estimate the average emotion by extracting emotional information from the different faces. However, are those estimations accurate? This is important during daily social interactions such as walking down the street, or, when giving a talk in front of an audience. Here, we examined whether our participants’ personal familiarity with the different identities within the group, as well as the emotional intensity of the different expressions in the group, biased estimations of the group’s emotion. In a series of studies, we show that when all the identities are unfamiliar, the presence of any high intensity emotional face biased crowd perception towards its emotion. However, when a familiar identity was present in the ensemble, perception was biased towards the familiar face’s emotion regardless of its intensity. These findings have important implications for how we might be biased in the judgements we make about a group’s overall emotional state.

## Ensemble perception

Successful social situations often require us to accurately track other people’s emotional states, and we are generally good at doing this (Keltner & Haidt, 1999; Olsson & Ochsner, 2008). However, people are rarely seen outside of any context (Barrett et al., 2011)—in other words, we rarely see faces on their own (as examined in traditional face perception studies); instead, we usually encounter faces as elements within visually rich environments (Soderberg & Sherman, 2013; Whitney & Yamanashi Leib, 2018). Furthermore, our ability to accurately perceive a complex scene is limited by finite attentional resources (Cavanagh & Alvarez, 2005; Dux & Marois, 2009; Simons & Levin, 1997). To deal with this, it has been suggested that the visual system extracts summary statistics about a scene (Oliva & Torralba, 2006). In ensemble perception, the mean information about a visual feature is extracted from a set of stimuli that vary along one or more feature dimensions through averaging (Ariely, 2001; Haberman et al., 2009; Oliva & Torralba, 2006; Whitney & Yamanashi Leib, 2018). Ensemble perception has been reported for various low-level visual features such as size (Ariely, 2001), brightness (Bauer, 2009), hue (Maule & Franklin, 2015; Webster et al., 2014), colour (De Gardelle & Summerfield, 2011), and orientation (Dakin & Watt, 1997; Parkes et al., 2001).

Ensemble perception has also been reported for the perception of higher-level visual features such as facial properties, including emotional expression (Haberman & Whitney, 2007, 2009), attractiveness (Walker & Vul, 2014), age (Awad et al., 2020), gender (Haberman & Whitney, 2007), and identity (de Fockert & Wolfenstein, 2009; Neumann et al., 2013). Haberman and Whitney (2009) presented participants with ensembles of faces varying in emotional intensity (e.g., the faces were morphed in different ratios between happy and sad) that were followed by a test face. Participants judged whether the test face had been a member of the previously viewed ensemble, and also whether the emotion of the test face appeared happier or sadder than the average of the ensemble. Although participants were unable to determine whether the test face had been in the original ensemble, they were able to correctly judge its emotional state compared with the average of the ensemble. Thus, the authors argued that participants retained little information about the individual members of the ensemble but possessed a good representation of the average emotion of the ensemble. However, in this study, the faces in the ensemble were all images of the same person whose expression varied in emotional intensity. Griffiths et al. (2018) also examined how the average facial expression of the ensemble influenced judgements of individual faces. Participants judged the emotional intensity of target faces against that of ensembles (that contained different identities with different emotional intensities) and found, consistent with Haberman and Whitney (2009), that judgments of the emotional intensity for individual faces were biased towards the group mean expression intensity.

During social interactions, we perceive groups of faces that vary on many dimensions, including identity, age, gender, race, attractiveness, and emotional expressions. Thus, an unresolved question in ensemble perception literature is what is averaged when we encounter such a diverse and visually rich group of faces (Bruce & Young, 1998; Haxby et al., 2000). Indeed, there is an ongoing debate about the way in which people compute these group averages. Some researchers suggest ensemble perception occurs by encoding all of the items in the group in a distributed manner (Baek & Chong, 2020), while others argue that ensemble perception occurs by sampling a subset of items (Allik et al., 2013; Maule & Franklin, 2015), with participants preferentially sampling the most salient items (Goldenberg et al., 2021; Kanaya et al., 2018; Sweeny et al., 2013). This debate about how ensemble representations are computed may be particularly relevant when it comes to people’s perception of groups of faces, which are both socially salient and visually complex objects.

For example, a recent study by Goldenberg et al. (2021) found that people attend to faces exhibiting intense emotions in the ensemble, generating a “crowd amplification effect,” where the ensemble’s emotional expression appears more intense than it is. The authors propose that attentional biases to emotional faces with extreme intensities in the ensemble result in the overreliance of those facial expressions when computing the average. They find that this effect increases with slower attentional disengagement or exposure time, both attributed to participants’ looking more at the intense emotional faces. The authors also found that participants who displayed the highest amplification effect also had increased fixation durations on emotional faces. However, the authors used same identity faces, so it is unclear how the visual system combines information about faces in heterogeneous groups. Here, we examine this by investigating the influences of emotional intensity and familiarity on ensemble perception of a group of faces.

## Facial expressions

The preferential processing of emotional stimuli over neutral stimuli is well documented (Dolan, 2002). For example, emotional faces produce an increase in the small voltages in the brain structure, referred to as an event-related brain potential (ERP; Blackwood & Muir, 1990) relative to neutral faces (Eimer & Holmes, 2007). Furthermore, some research suggests that negative emotional stimuli can sharpen the focus of selective attention (e.g., reduce attention to other, nonsignificant stimuli) due to their potential value in signalling benefit or danger (Fenske & Eastwood, 2003; Posner, 1980), while positive emotional stimuli can broaden the scope of attention (Fredrickson & Branigan, 2005; Srinivasan & Hanif, 2010).

It remains unclear whether some facial emotions influence perception more than others—for example, the “face in the crowd” effect. Some studies suggest an “anger superiority effect,” where threatening or angry faces are detected more efficiently than happy or nonthreatening faces in a crowd of faces, possibly reflecting the need to quickly locate and identify potentially threat (Horstmann & Bauland, 2006; Pinkham et al., 2010). Others argue for a “happy superiority effect,” which reflects a search bias towards happy faces and is thought to reflect the less ambiguous communicative intent of happy faces (Becker et al., 2011). Here, we were interested in investigating whether emotional intensity and familiarity influence perception independently of the valence (or polarity) of the expression and therefore used both positive (happy) and negative (angry) facial expressions.

## Face familiarity

Familiar faces are believed to be processed differently than unfamiliar faces (Haxby & Gobbini, 2011; Ramon & Gobbini, 2018), with recognition of familiar faces being significantly higher than recognition of unfamiliar faces (Bruce et al., 2001; Burton et al., 2015; Ramon & Van Belle, 2016). Familiar faces are also more resistant to the attentional blink than are unfamiliar faces. For example, Gobbini et al. (2013) used a continuous flash interocular suppression task, which renders the faces invisible, and measured face detection time for personally familiar faces (e.g., friends and colleagues) and unfamiliar faces. They found that personally familiar faces broke through the suppression ∼90-ms faster than unfamiliar faces. Thus, across different types of tasks, familiar faces appear to be processed differently from unfamiliar faces. In addition to enhanced visual encoding, some studies have shown that familiar faces also activate brain regions associated with storing and processing information about a person (such as semantic, episodic, and emotional information). This suggests that the brain processes familiar and unfamiliar faces in different neural structures as well (for a review, see Natu & O’Toole, 2011).

Some studies have examined whether the differences in perception of familiar and unfamiliar faces may be associated with differences in how people look at these types of faces (Althoff & Cohen, 1999; Kita et al., 2010; Stacey et al., 2005). Earlier studies suggested a difference in the overall number of fixations on familiar and unfamiliar faces (although in those experiments each face is viewed alone), reflecting differences in how participants scanned the faces. Specifically, subjects were more likely to fixate on the internal facial features (e.g., eyes) of a familiar face compared with an unfamiliar face (Althoff et al., 1999). However, more recent research has found that overall fixations and scanning strategies do not differ between familiar and unfamiliar faces, and that differences in perception arise at a later stage of processing (Kita et al., 2010).

## The current study

Although people can extract information about the average of a group of faces (Alvarez & Oliva, 2008; Whitney & Yamanashi Leib, 2018), it is unclear how different faces, with varying levels of saliency, contribute to this average representation. In this study, we investigate whether participants are influenced by the emotional intensity and identity of the test faces when making judgments about the average emotion of a group of faces, or whether some faces have a greater influence on their average judgments. We used faces that could vary in emotional intensity (high/low), valence (happy/angry) and personal familiarity (yes/no). We also examined whether any biases attributed to familiar faces in the ensemble were the result of differences in perceived expression intensity between familiar and unfamiliar faces. Based on the “angry superiority effect” and the “happy superiority effect,” we expected that the perception of ensembles containing different facial expressions would be biased toward the high intensity expressions in the ensemble, regardless of their valence (Experiment 1). Additionally, based on reports of preferential processing for familiar faces, we expected that perception of the ensemble would be biased towards the familiar face’s emotional expression (Experiment 2). We did not expect to find a familiarity effect in Experiment 1, because we used faces that were unfamiliar to all our participants. We did this as a control to ensure that any differences found in Experiment 2 were not due to individual differences in their ability to average groups of emotional faces.

## Experiments 1 and 2: General methods

### Participants

To evaluate the appropriate sample size for the study, we used data from a recent study on group categorization that provided initial evidence for the occurrence of estimation bias (Goldenberg et al., 2021). In that study, 30 participants completed the experiment.

In this study, 28 female participants were divided into two groups: (a) familiar group (*n* = 14; participants were familiar with some of the faces, mean age = 26.57 years, *SD* = 3.57 years); and (b) an unfamiliar group (*n* = 14; participants were unfamiliar with all of the faces, mean age = 24.64 years, *SD* = 3.13). These two groups took part in both experiments. All participants had normal or corrected-to-normal vision. The experiment was approved by the ethics board of Queen Mary University of London (reference number QMREC2239). Participants gave written informed consent to take part in the study and received monetary compensation for their participation. The familiar group consisted of graduate students from the Psychology department at Queen Mary University of London and had known the people in the photographs for at least 1 year. The unfamiliar group were psychology undergraduate students and staff members from another department in Queen Mary University of London.

### Apparatus

The experiment took place in a dimly lit room. Stimulus presentation and response collection was controlled by a DELL PC running MATLAB software (The MathWorks Ltd., Natick, MA, USA) with Psychtoolbox (Brainard, 1997). The stimuli were presented on an Iiyama Vision Master PRO 520 monitor (1,600 × 1,200 pixels, 60-Hz refresh rate). At viewing distance of 57 cm, 1 pixel subtended 1.5 arcmin. A chin rest was used to ensure constant viewing distance to the monitor, and a Tobii 4C eye tracker was used to record participants’ eye movements during the experiments.

### Stimulus generation

**Fig. 1:**
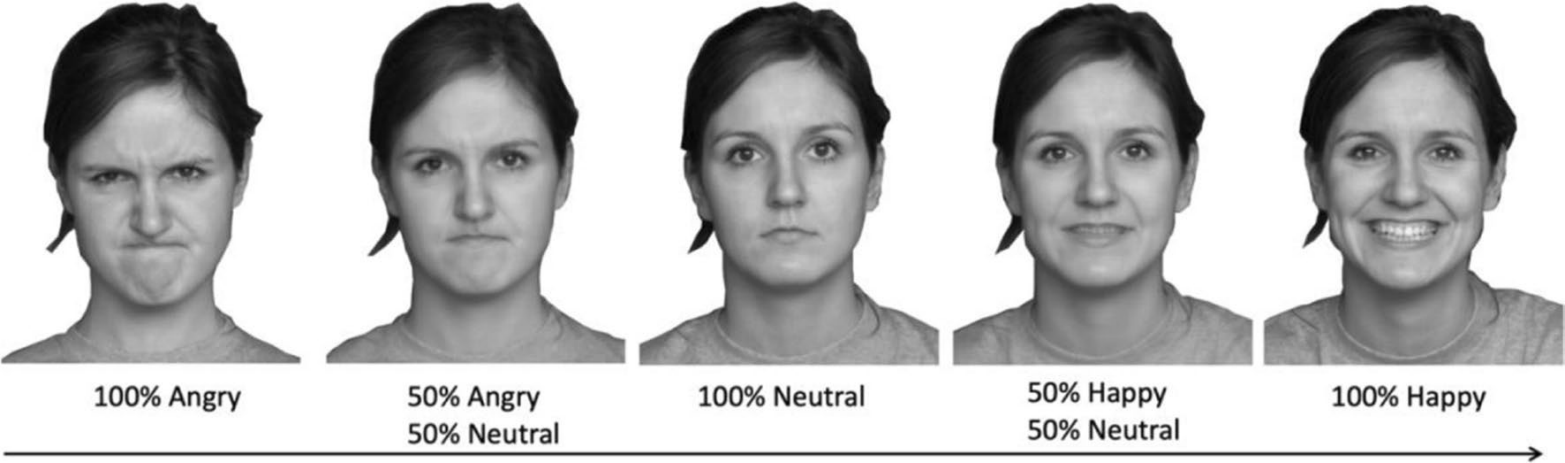
Sample morphed facial expressions from angry to happy (going through neutral) in steps of 50% morph units for the faces used in the ensemble. The test faces were morphed in steps of 20% morph units on the angry–neutral–happy continuum. For anonymity purposes, we illustrate the procedure with faces taken from FACES database (Ebner et al., 2010)

To create the stimuli, the researchers took photographs of 20 confederate female students’ faces using a Nikon D5000 digital camera. Ten of the confederate faces were labelled as unfamiliar stimuli (unfamiliar to all participants) and were used in both experiments. Ten were labelled as familiar stimuli (familiar to the familiar participants group only) and were only used in Experiment 2. The confederate students stood against a white wall with overhead neon lighting. To create different intensity expressions, each student was instructed to make a happy, angry, and neutral facial expression. For terminology purposes, we refer to the unmorphed happy and angry expressions as high intensity. We then created two low-intensity morphed expressions using FantaMorph, one low-intensity happy consisting of a 50% happy and 50% neutral morph, and one low-intensity angry consisting of a 50% angry and 50% neutral morph (see Fig. 1). This resulted in a total of five facial expressions per identity, or 100 test expressions in total. After taking each photograph, we asked the confederate if it was an accurate portrayal of the emotion they were expressing. If they said no, we took another photograph until they were satisfied.

#### Ensembles

Each ensemble consisted of four facial expressions from four different identities. The identities were randomly selected and then randomly positioned within the ensemble on each trial. The faces were presented around the centre of the screen in square formation, with each face subtending 9**°** **×** 11**°**. All stimuli were processed with Adobe Photoshop to have a transparent background around the face and neck area and were presented in grayscale and matched in RMS contrast.

#### Test faces

To measure the emotion of the ensemble, a test facial expression was presented immediately after the ensemble, and participants had to judge whether it looked happier or angrier than the ensemble average. To create the range of test facial expressions, for each confederate identity, the researchers created morphs between the confederate’s happy and angry expressions, going through neutral, in steps of 20%, which resulted in 11 test faces. The test facial expressions subtended 9**°** **×** 11**°** and were presented in the centre of the screen. The test facial expressions were always unfamiliar to both groups of participants.

### Behavioural task procedure

**Fig. 2:**
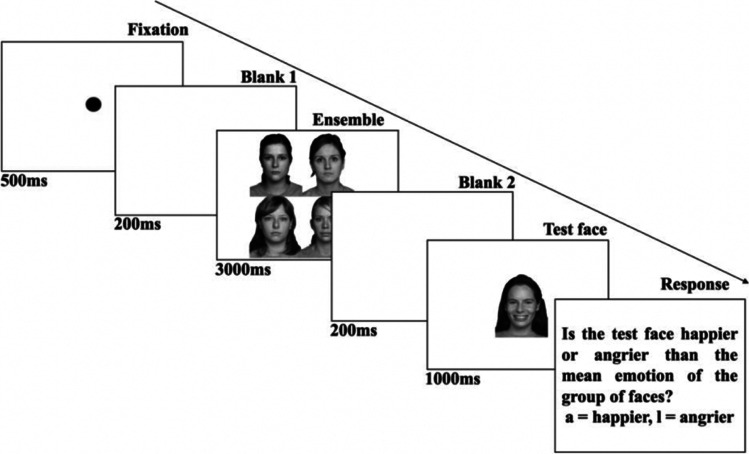
Timeline of a single trial showing an all-neutral condition (all faces expressing a neutral expression), followed by a high intensity happy (100% happy morph) test face

We used Haberman and Whitney’s (2007, 2009) paradigm to measure ensemble emotion perception. Participants viewed an ensemble of four different identity faces, followed immediately by a test facial expression, and indicated with a key press whether the test facial expression looked happier or angrier than the average of the ensemble. Each trial began with a 500-ms fixation point (10 pixels diameter fixation dot), followed by a blank screen for 200 ms. The ensemble was then presented for 3,000 ms, followed immediately by a blank screen for 200 ms. Participants were presented with a test facial expression for 1,000 ms and then prompted to indicate whether the test facial expression was happier or angrier than the average emotion of the ensemble (by pressing an “A” key for happier and “L” key for angrier). Key-press responses could only be recorded once the test face had been extinguished. Figure 2 illustrates a trial sequence.

The identities for the ensemble and test faces were randomly selected with three constraints: (1) no two faces in any ensemble were the same identity, (2) no test face was an identity in the ensemble of faces for the same trial, and (3) test faces were always of unfamiliar confederates. Experiments 1 and 2 followed this procedure, with 198 trials in Experiment 1 and 264 trials in Experiment 2. Test facial expressions had 11 different expression levels, with each level repeated six times per condition. There were three ensemble conditions in Experiment 1 and four ensemble conditions in Experiment 2 (see Tables 1 and 2) that were randomly interleaved across six blocks. Each ensemble condition in each experiment was repeated for 66 trials (each condition was tested with 11 test facial expressions × 6 repeats).

**Table 1:** Different ensemble conditions in Experiment 1

Ensemble	Face 1	Face 2	Face 3	Face 4
All neutral	100% Neutral	100% Neutral	100% Neutral	100% Neutral
High-intensity happy face	100% Happy	50% Angry	50% Angry	100% Neutral
High-intensity angry face	100% Angry	50% Happy	50% Happy	100% Neutral

**Table 2:** Experiment 2 conditions (the familiar face intensity is indicated in bold)

	Face 1	Face 2	Face 3	Face 4
High-intensity familiar face	*100% High intensity (familiar)*	50%low intensity (unfamiliar)	50% low intensity (unfamiliar)	100% Neutral (unfamiliar)
Low-intensity familiar face	100%High intensity (unfamiliar)	*50% low intensity (familiar)*	50% low intensity (unfamiliar)	100% Neutral (unfamiliar)

## Experiment 1

### Aims and design

This experiment had two main aims: (A1) to investigate whether the averaging of the ensemble would be biased towards the emotion of the high-intensity emotional faces and (A2) to ensure that performance was similar in the two participant groups when tested with unfamiliar faces.

**Fig. 3:**
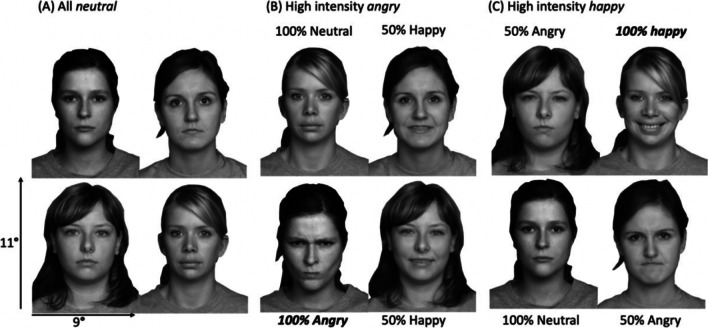
Sample ensemble conditions in Experiment 1. **A** An ensemble composed of neutral faces. **B** An ensemble composed of a high-intensity angry expression, two low-intensity happy faces, and a neutral face. **C** An ensemble composed of a high-intensity happy expression, two low-intensity angry expressions, and one neutral expression. Face identities and positions were randomized on every trial. For illustrative purposes, only and to maintain anonymity for our confederates, the images displayed are from FACES database (Ebner et al., 2010) and were not used in the experiments

To address A1, we created three different ensemble conditions with mixed facial expressions and intensities (Fig. 3). These were designed to test whether ensemble perception was biased by high-intensity emotions (Ensembles 1 and 2) and to test participants’ ensemble perception for neutral (Ensemble 3 all faces neutral) as a baseline condition. Table 1 summarizes the different ensembles. To quantify the influence of emotions/intensities, we measured bias in the different ensembles. To address A2, we compared performance between the two groups across the different conditions (familiarity effect).

### Behavioural data analysis

**Fig. 4:**
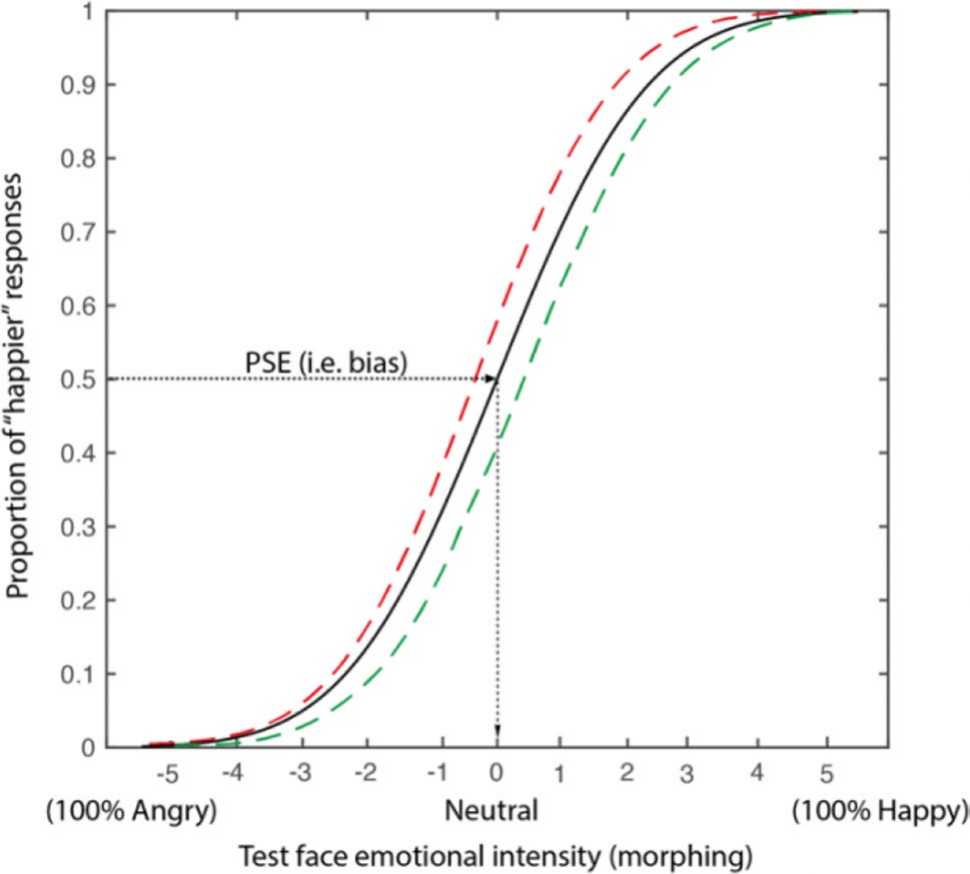
Example psychometric functions plotting the proportion of times the facial emotion appeared “happier” than the ensemble emotion, as a function of test face expression intensity. The black psychometric function illustrates no bias, the red dashed function illustrates an angry bias, and the dashed green function a happy bias for perception of the ensemble. The PSE (that we refer to as “neutral”) corresponds to the morph level where the test facial expression was perceived to be neither happier nor angrier than the average emotion of the ensemble. (Colour figure online)

To extract participants’ bias across the different ensemble conditions, we estimated the point of subjective equality (PSE). This represents the test facial expression morph level where participants judged it to appear happier than the ensemble in 50% of the trials (or equally, to appear angrier than the ensemble in 50% of the trials), meaning that its emotional expression appeared identical to the average emotion of the ensemble. To obtain the PSE, we plotted the proportion of times the test facial expression was judged to be “happier” than the ensemble as a function of the test facial expression morph level—that varied in 11 steps between angry and happy, where the midpoint was the neutral face—and extracted the test morph level corresponding to the 50% happier response. Therefore, a PSE of 0 would indicate that the participant perceived the average emotion of the ensemble to be similar to the neutral test facial expression. A PSE to the right of 0 would indicate that a participant judged a (slightly) morphed happy test facial expression to have the same emotion as the ensemble, therefore the ensemble was perceived as happy. Figure 4 illustrates three sample psychometric functions when the participant displayed no bias (black curve), a happy bias (green curve), and an angry bias (red curve). Note that the psychometric functions here relate to the previously presented *ensemble* in the trial, not the test face itself.

### Eye-tracking data analysis

We used a Tobii 4C eye tracker to measure how participants looked at the faces in the ensemble when performing the averaging task. Raw eye position data were parsed by the eye tracker software’s standard experimental setting. We defined 4 areas of interest (AOIs) subtending 9**°** × 11**°**, that entirely covered the faces (plus their background) and extracted fixations within each AOI on every trial. For each of the ensemble conditions, we measured the proportion of fixations (lasting at least 100 ms) on the different faces across all trials. The proportion of fixations on the different expressions were then analyzed by means of separate one-way analyses of variance (ANOVAs; for each ensemble condition) in Experiment 1 with the faces’ emotional expression and intensity (four levels) as the within subjects’ factor. A similar analysis was performed in Experiment 2, but with participants’ familiarity as between subjects’ factors.

### Results and discussion

#### Behavioural results

**Fig. 5:**
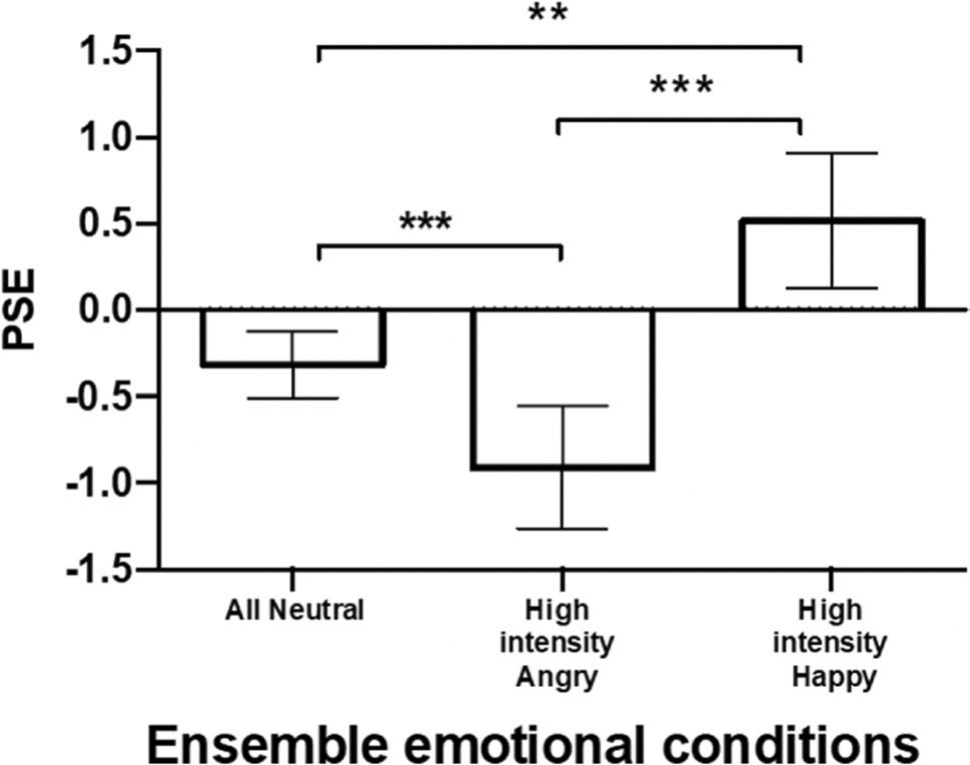
High-intensity expressions bias ensemble perception. PSEs in the different ensemble conditions (see Table 1) averaged across the two participants’ groups. Error bars represent 95% confidence interval (CI). ***p ≤ 0.001, **p ≤ 0.01

To examine the relationship between the different emotional expressions and intensities on ensemble emotion perception, we ran a two-way repeated-measures mixed ANOVA on the average PSE scores. The within-subjects factor was ensemble condition (three levels), and the between-subjects factor was familiarity (or participants’ group; two levels). Mauchly’s test indicated that the assumption of sphericity had been violated for the ensemble conditions; therefore, Greenhouse–Geisser corrections are reported. We found a significant main effect of the different ensembles on the PSE scores, *F*(1.23, 31.99) = 18.61, *p* < 0.001, ${\eta }_{p}^{2}$ = 0.42 (Fig. 5), but no significant effect of familiarity. *F*(1, 26) = 0.24, *p* > 0.050, ${\eta }_{p}^{2}$ = 0.01, and no significant interaction between ensemble conditions and familiarity, *F*(1.23, 31.99) = 0.89, *p* > 0.050, ${\eta }_{p}^{2}$ = 0.03. Therefore, the two groups did not differ in their perception of ensemble emotion using unfamiliar stimuli. To further analyze the main effect of the ensemble emotion expression, we ran a series of Bonferroni-corrected paired *t* tests (Fig. 5). Notably, we also find that the PSE score in the all-neutral condition (*M* =  − 0.31, *SD* = 0.49) is found to show a slight negative bias compared with a PSE score of 0 (no bias), a statistically significant difference of − 0.31 (95% CI [− 0.51, − 0.1]), *t*(27) =  − 3.422, *p* < 0.001, suggesting a slight negative bias in general.

#### Eye-tracking results

Since there was no effect of familiarity on PSE, we averaged eye-tracking data across the two participant groups and performed three separate one-way repeated-measures ANOVAs on the proportion of fixations directed towards the different faces for each ensemble condition (Table 1). We found no significant effect of emotional expression or intensity on the proportion of fixations for any of the ensemble conditions—Ensemble 1, no significant main effect of face, *F*(3, 81) = 0.21, *p* > 0.050, ${\eta }_{p}^{2}$ = 0.01; Ensemble 2, no significant main effect of face, *F*(3, 81) = 0.94, *p* > 0.050, ${\eta }_{p}^{2}$ = 0.03; Ensemble 3, no effect of face, *F*(3, 81) = 1.73, *p* > 0.050, ${\eta }_{p}^{2}$ = 0.06.

To summarize, Experiment 1 measured emotion ensemble perception in a group of different identity faces and examined how different emotional expressions (happy or angry) and intensities (high or low) influenced performance. Consistent with previous reports, we found evidence for ensemble perception of emotional expressions (Whitney & Yamanashi Leib, 2018). However, we also found that ensemble perception is influenced by the presence of a high-intensity emotional expression, with participants overestimating the emotional intensity of the ensemble in the same direction as the high-intensity emotional face. We found no difference in fixations between the different facial emotions and intensities, suggesting that the behavioural biases might be due to ensemble perception mechanisms rather than differences in face scanning. This experiment revealed, as expected, that there were no differences between the two participants groups in their ensemble perception with unfamiliar stimuli.

## Experiment 2

In Experiment 2, we examined whether the presence of a familiar face affects ensemble emotion perception by creating ensembles where one of the four faces was personally familiar (e.g., a friend or colleague) to participants from the familiar participants group only. Participants from the unfamiliar participants group viewed the same ensemble, but all the faces were unfamiliar to them. We used the same identity unfamiliar faces as in Experiment 1.

### Methods and stimuli

The methods were identical to those used Experiment 1 (as explained in the General Methods section), except for the addition of new facial stimuli. In addition to the 10 confederates used to create unfamiliar stimuli in Experiment 1, we used 10 additional confederates in Experiment 2 that were known only to the participants in the familiar participants group.

#### Procedure and design

The procedure was identical to that used in Experiment 1. There were 264 experimental trials in total. The test faces were drawn from 11 test levels, with each level repeated six times per condition. The experiment had two ensemble conditions, which are summarized in Table 2.

#### Eye-tracking data analysis

Eye tracking and data analysis were identical to those used in Experiment 1. However, since differences in perceived ensemble emotion in Experiment 1 were driven by the intensity of facial emotion rather than the type of emotion (angry or happy), we combined angry and happy familiar conditions (of the same emotion intensity) together (by flipping the PSE’s sign in the angry conditions), resulting in a high intensity familiar face condition (regardless of emotion type), and a low intensity familiar face condition (regardless of emotion type).

### Results and discussion

#### Behavioural data

**Fig. 6:**
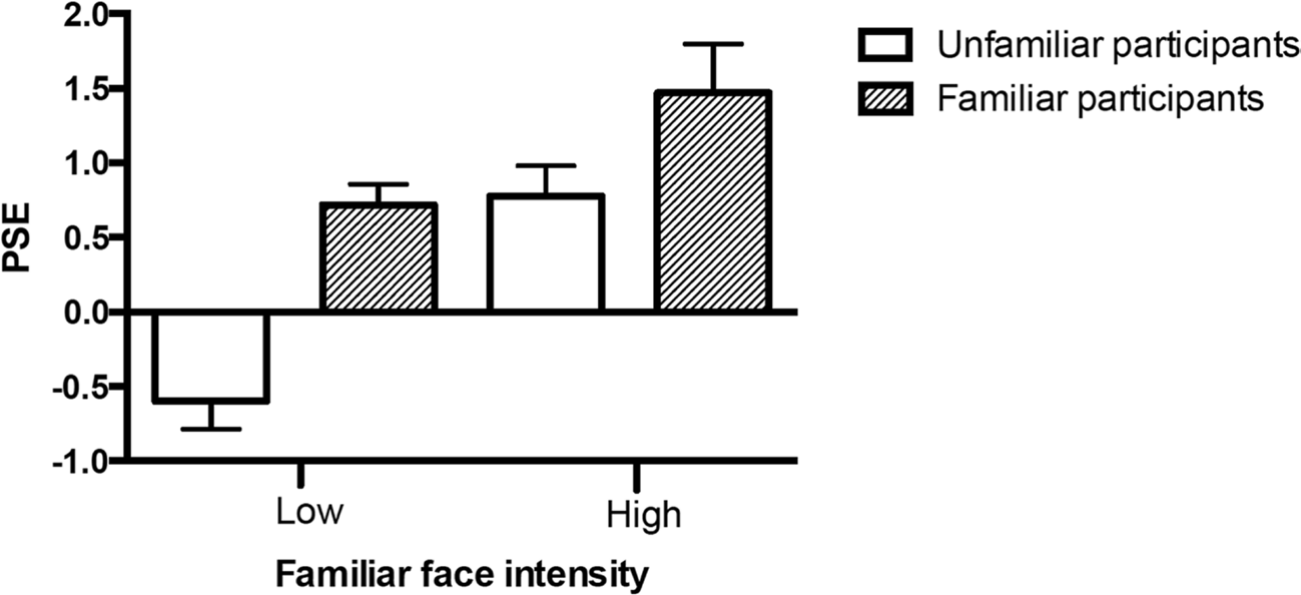
Experiment 2 averaged PSEs as a function of the emotional expression and intensity of the familiar faces in each ensemble. Familiar participants’ results are shown in hashed bars and unfamiliar participants in open bars. Error bars represent 95% confidence interval (CI)

To examine the influence of face familiarity on ensemble emotion perception, we ran a two-way mixed ANOVA on the PSEs, with ensemble condition as the within-groups factor (two levels; low- vs. high-intensity familiar face) and participants’ group as the between-subjects factor (two levels; familiar vs. unfamiliar participants). We found a significant main effect of ensemble condition, *F*(1, 26) = 18.23, *p* < 0.001, ${\eta }_{p}^{2}$ = 0.41, indicating that performance with ensembles with low intensity familiar faces (*M* = 0.09, *SD* = 1.00) was less biased overall than ensembles with high-intensity familiar faces (*M* = 1.10, *SD* = 1.00). We also found a significant main effect of familiarity, *F*(1, 26) = 24.39, *p* < 0.001, ${\eta }_{p}^{2}$ = 0.48, indicating that familiar (*M* = 1.13, *SD* = 1.06) and unfamiliar participants (*M* = 0.61, *SD* = 0.91) had significantly different biases. However, there was no significant interaction between ensemble conditions and familiarity, *F*(1, 26) = 1.74, *p* > 0.050, ${\eta }_{p}^{2}$ = 0.06. This is particularly apparent in ensembles with low-intensity familiar faces, as participants’ performance is in a different direction, as illustrated in Fig. 6.

#### Eye-tracking data

##### Low-intensity familiar face

**Fig. 7:**
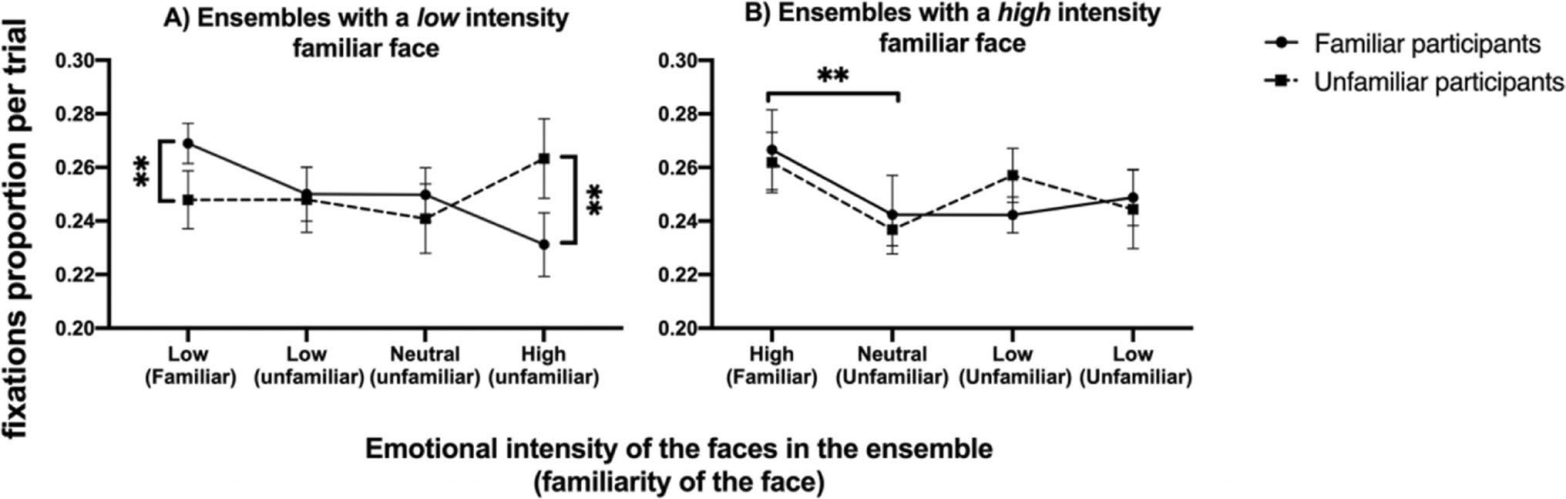
Eye-tracking results for familiar and unfamiliar participants. **A** Proportion of overall fixations when the familiar face in the ensembles is low intensity. **B** Proportion of overall fixations when the familiar face in the ensembles is high. Error bars are 95% confidence intervals for the means. **p ≤ 0.01

To measure the proportion of fixations on the different faces in an ensemble that contained a *low-intensity familiar face,* we ran a two-way mixed ANOVA, with intensity of face type as the within-subject factor (four levels: familiar low intensity; unfamiliar high intensity, unfamiliar low intensity, unfamiliar neutral) and participants’ group as the between-subjects factor (two levels; familiar vs. unfamiliar participants). We do not find a main effect of face type, *F*(3, 78) = 1.80, *p* > 0.050, ${\eta }_{p}^{2}$ = 0.07. There was also no main effect of participants’ group, *F*(1, 26) = 0.14, *p* > 0.050, ${\eta }_{p}^{2}$ = 0.05. However, there was a significant interaction, *F*(3, 78) = 6.92, *p* < 0.01, ${\eta }_{p}^{2}$ = 0.21 (Fig. 7A). To further analyze this, we ran paired *t* tests for different face types between the two groups and found a significant difference, *t*(13) = 3.85, *p* < 0.050, between the proportion of fixations on the low-intensity familiar face, revealing that familiar participants fixated more on the low-intensity familiar face (*M* = 0.27, *SD* = 0.012) than unfamiliar participants (*M* = 0.24, *SD* = 0.18). We also found a significant difference, *t*(13) =  − 3.16, *p* < 0.050, between the proportion of fixations on the unfamiliar high-intensity face between familiar (*M* = 0.23, *SD* = 0.25) and unfamiliar participants’ group (*M* = 0.26, *SD* = 0.025).

##### High-intensity familiar face

We measured the proportion of fixations on the different faces of the ensemble using a two-way mixed ANOVA, with emotional intensity of face type as the within-subject factor (four levels: high-intensity familiar face, low-intensity unfamiliar face, low-intensity unfamiliar face, neutral unfamiliar face) and participants’ group (two levels; familiar vs. unfamiliar participants) in an ensemble which contained a *high-intensity familiar face.* We found a significant main effect of emotional intensity, *F*(3, 78) = 5.63, *p* < 0.005, ${\eta }_{p}^{2}$ = 0.18, but no main effect of participants’ group, *F*(1, 26) = 0.13, *p* > 0.050, ${\eta }_{p}^{2}$ = 0.01, and no significant interaction, *F*(3,78) = 1.26, *p* > 0.050, ${\eta }_{p}^{2}$ = 0.05. To further analyze the main effect of emotional intensity, we ran a series of Bonferroni-corrected paired *t* tests, and the only significant difference, *t*(27) =  − 3.54, *p* < 0.050, was between the high-intensity emotional face (*M* = 0.26, *SD* = 0.02) and the neutral face (*M* = 0.23, *SD* = 0.02), as illustrated in Fig. 7B.

To summarize, we found that participants who were familiar with a face in the ensemble fixated more on the low-intensity familiar face and were biased towards its emotional expression more than participants who were unfamiliar with the face. This is particularly interesting as this occurred even in the presence of a high intensity (nonfamiliar) face. This suggests that participants relied more on information conveyed by familiar faces, even when the familiar face was not the most emotionally salient one in the ensemble. This indicates that participants may place greater weight on information from familiar faces when making judgments about ensemble emotions. There were no differences between groups when the familiar face was high intensity.

## Experiment 3: Emotional intensity ratings

The experiment aimed to examine whether the perceptual biases observed with familiar faces in Experiment 2 were due to differences in the perceived intensity of familiar faces compared with unfamiliar faces. If low-intensity familiar faces appeared more intense than high-intensity unfamiliar faces, this could challenge the interpretation that the biases are solely a result of processes related to ensemble emotion perception.

### Methods

#### Participants

Twenty-two female participants took part in this experiment. The 11 familiar participants were the same (mean age = 26.92 years, *SD* = 3.57 years) as in Experiments 1 and 2, and 11 new unfamiliar participants (mean age = 29.55 years, *SD* = 5.39 years) were recruited online.

#### Procedure

On every trial, participants were asked to rate the intensity of individual facial expressions on a scale from 0 to 100. Each participant rated each of the 40 individual facial expressions used in Experiment 2 once (10 familiar identities each across the four Morph levels). Images were presented in greyscale and matched for RMS contrast. Given constraints of the COVID-19 pandemic, the experiment was conducted online rather than in the lab. Images were randomized and uploaded to a Qualtrics online survey (Qualtrics, Provo, UT, 2017). Each trial was self-paced, and participants entered their responses using a slider scale.

### Results

**Fig. 8:**
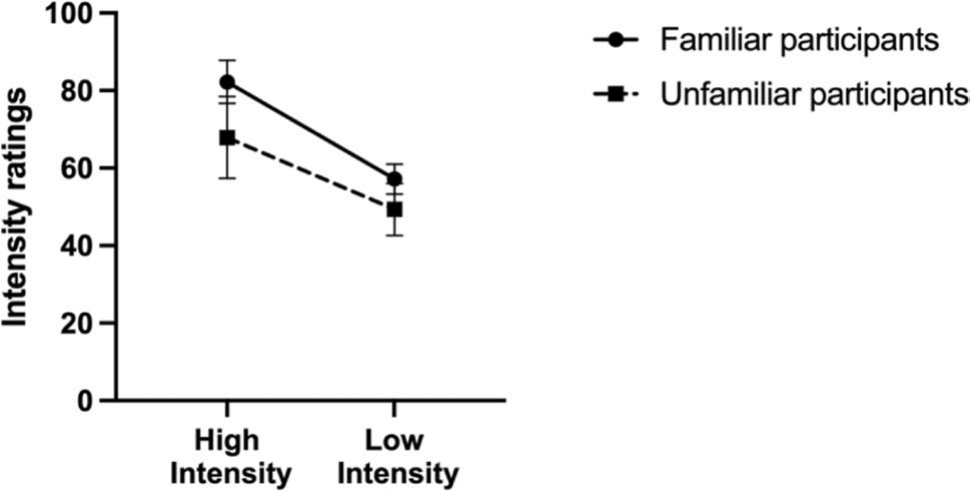
Familiar face emotional intensity ratings averaged across the different participants’ groups (familiar and unfamiliar participants) for different emotional intensities (high and low). Error bars are 95% confidence intervals for the means

As in the previous experiments, we combined same intensity angry and happy familiar conditions together, resulting in two intensity conditions (high and low) with familiar faces. We ran a two-way repeated-measures ANOVA with expression intensity (two levels: high intensity, low intensity), as the within-subject factor, and participants’ group (two levels; familiar vs. unfamiliar participants) as the between-subjects factor. We found a significant main effect of expression intensity, *F*(1, 20) = 89.41, *p* < 0.001, ${\eta }_{p}^{2}$ = 0.81. To further analyze this main effect, we ran Bonferroni-corrected paired *t* tests and found significant differences between the different facial expression intensities, *t*(21) = 8.89, *p* < 0.001, where high-intensity facial expressions (*M* = 74.07, *SD* = 14.81) were rated as more intense than low-intensity facial expressions (M = 53.27, *SD* = 8.90; Fig. 8). There was also a main effect of participants’ group *F*(1, 20) = 9.14, *p* < 0.050, ${\eta }_{p}^{2}$ = 0.32; where familiar participants rated familiar facial expressions (*M* = 69.72, *SD* = 6.81) as more intense than unfamiliar participants (*M* = 57.64, *SD* = 11.13; Fig. 8). There was no significant interaction between face type and familiarity, *F*(1, 20) = 3.73, *p* > 0.050, ${\eta }_{p}^{2}$ = 0.16.

### Discussion

We examined emotion ensemble perception using faces with different emotional expressions and intensities, some of which were familiar to participants. Consistent with previous reports, we found ensemble perception for emotional expressions (Haberman & Whitney, 2007, 2009). Additionally, we found that the presence of a single high-intensity emotional face had a greater impact on ensemble perception than lower intensity emotional faces (Goldenberg et al., 2021; Mihalache et al., 2021). Specifically, we observed a systematic bias for the ensemble to be perceived as angrier when there was a high-intensity angry face present, and to be perceived as happier when there was a high-intensity happy face present. Interestingly, when a familiar face was present in the ensemble, it biased ensemble perception towards its expression, regardless of its intensity. We discuss below possible mechanisms involved in these processes.

Our aim was to investigate how different facial expressions in an ensemble influenced participants’ perception of the average emotion. This is important because ensemble perception is traditionally thought to overcome the limited capacity of the visual system by not requiring detailed perception of individual items in the ensemble (Chong & Treisman, 2005; Corbett & Oriet, 2011; Ward et al., 2016), although other reports indicate that some items do bias the ensemble average (de Fockert & Marchant, 2008; H. Li et al., 2016; V. Li et al., 2017; Neumann et al., 2018). These previously reported biases affected by an individual item have been attributed to focused attention (de Fockert & Marchant, 2008; Goldenberg et al., 2021), primacy and recency effects (Hubert-Wallander & Boynton, 2015) or the item’s value (Dodgson & Raymond, 2020) rendering them more salient and therefore more likely to influence ensemble perception (Kanaya et al., 2018). Our findings further support the idea that not all items affect ensemble emotion perception equally, and that familiarity and emotional intensity play an important role in generating summary statistics.

#### Role of facial emotional intensity in ensemble perception

What might explain the changes in perceived average emotion of the ensemble when faces with different emotional expressions and intensities are present? One possibility is that attention may bias ensemble perception. For example, it has been suggested that an attentional bias towards highly emotional faces might lead to a corresponding bias in estimating the average ensemble emotion due to a stronger visual memory of the highly emotional faces, which in turn shapes the estimation of the average (Brady & Alvarez, 2011; Goldenberg et al., 2021). Indeed, previous research has shown that in certain cases, perception can be biased in favour of emotional faces that contain critical information by automatically capturing attention even when they are task irrelevant (Becker et al., 2011; Fenske & Eastwood, 2003). Although attention can be deployed independently of where the eyes are looking (Hunt et al., 2019; Mahon et al., 2018; Posner, 1980), our eye-tracking results showed no difference in the proportion of fixations between the different emotional intensity faces when the faces were of a high-emotional intensity. However, our paradigm differed from that of Goldenberg et al.’s (2021) in a few ways. Firstly, their ensembles were all the same identity faces, while our ensembles consisted of faces with different identities. Secondly, their ensembles conveyed the same emotional expression (but different intensities), while our ensembles consisted of a mix of emotional expressions and intensities. Therefore, our findings suggest that the observed behavioural differences might be due to preferential processing of emotional faces in ensemble perception rather than a greater fixation on high-intensity emotional faces in the ensemble.

Alternatively, or in tandem with an attentional explanation, visual working memory may be influencing performance. For example, the social relevance of happy and angry faces might prompt memory for these emotional faces more so than for neutral faces. It has been shown that smiling (happy) faces facilitate memory through increased activation of brain regions associated with reward (Tsukiura & Cabeza, 2008). Similarly, Jackson and colleagues (2014) found that angry expressions strengthened the encoding and maintenance of face identity in visual working memory, which may be related to increased activity in the basal ganglia (Jackson et al., 2008, 2014). This is also supported by recent findings that memory for highly emotional stimuli is increased compared with mildly emotional or neutral stimuli, which is hypothesized to be due to the enhanced metacognitive feelings triggered by increasing emotional intensity (Meng et al., 2017; Schaefer et al., 2011). Thus, if working memory affects ensemble perception, enhanced memory of specific expressions might underlie the biases we report.

#### Relevance of face familiarity in ensemble perception

We also find that face familiarity biases perception of the ensemble average emotion. In this case, when a familiar face is high in emotional intensity, it leads to a greater bias compared with when the high-emotional intensity face is unfamiliar to observers. Furthermore, when there is a low-intensity familiar face, perception is biased by the familiar face in the ensemble rather than the high-intensity (unfamiliar) face. If, as we found in Experiment 3, the perceived emotional intensity of the familiar face is higher than that of unfamiliar faces, this could account for a greater change in the bias. However, it is unclear whether perceived intensity difference is the only contributing factor to the observed biases. For example, we found a significant familiarity bias despite the approximately equal perceived intensity between *familiar low-intensity faces* and *unfamiliar high-intensity faces.* We might have expected the *familiar low-intensity faces,* and *unfamiliar high-intensity faces* to have cancelled each other out due to their approximately equal *perceived* intensity. In this case, participants would reach their final decision according to the emotional expression of the remaining two faces in the ensemble; a *low-intensity unfamiliar face* and a neutral face, and then we would have expected to see a smaller bias towards the emotional expression of the remaining low-intensity unfamiliar face compared with a high-intensity unfamiliar face, which is inconsistent with our results (Fig. 6). Alternatively, it is possible that the familiar face biases reflect prioritized perception of personally familiar faces (Gobbini, 2010; Gobbini & Haxby, 2007; Johnston & Edmonds, 2009; Ramon & Gobbini, 2018). This area of research suggests that people have a preferential processing bias for familiar faces, which may be due to the enhanced neural representations of these faces. For example, Natu and O’Toole (2011) suggest that in addition to improved visual coding, familiar faces also activate brain regions associated with the representation of semantic, episodic, and emotional information about the person. This allows people to process and interpret familiar faces more efficiently and accurately, which may be why they have a preferential processing bias for them. Their review of the neural processing of familiar and unfamiliar faces provides evidence to support this idea. Another study by Mrkva and Boven (2020) found that repeated exposure to the same stimulus influences its perception and evaluation by increasing its saliency and makes it stand out, consistent with our findings of increased perceived intensity.

We were also interested in examining whether this increased perceived intensity also influenced visual attention. Our eye-tracking results are largely consistent with earlier reports suggesting no differences in overall fixations on familiar and unfamiliar faces (Althoff & Cohen, 1999; Kita et al., 2010; Stacey et al., 2005), apart from low-emotional intensity familiar faces. In this case, familiar participants fixated the familiar face more than unfamiliar participants, possibly due to a combination of the ambiguity of low-intensity faces, and their social relevance, suggesting that this attentional bias to low-intensity familiar faces may have resulted from increased attention to those faces (Brady & Alvarez, 2011; Goldenberg et al., 2021). Therefore, it appears that some differences in scanning of the faces may have played a role in our low intensity results.

In conclusion, this study extends recent attempts to investigate the role of ensemble perception in processes that are important for social interactions and functioning. We found that participants could integrate visual information from multiple faces, but that this process was influenced by the emotional expression intensity of the individual faces and whether the faces were familiar to participants or not. It is likely that a number of processes underlie this effect, including changes in perceived intensity which may influence attention and memory processes.
